# mTOR signaling mediates resistance to tankyrase inhibitors in Wnt-driven colorectal cancer

**DOI:** 10.18632/oncotarget.18146

**Published:** 2017-05-24

**Authors:** Tetsuo Mashima, Yoko Taneda, Myung-Kyu Jang, Anna Mizutani, Yukiko Muramatsu, Haruka Yoshida, Ayana Sato, Noritaka Tanaka, Yoshikazu Sugimoto, Hiroyuki Seimiya

**Affiliations:** ^1^ Division of Molecular Biotherapy, Cancer Chemotherapy Center, Japanese Foundation for Cancer Research, Tokyo, Japan; ^2^ Department of Computational Biology and Medical Sciences, Graduate School of Frontier Sciences, The University of Tokyo, Tokyo, Japan; ^3^ Division of Chemotherapy, Faculty of Pharmacy, Keio University, Tokyo, Japan

**Keywords:** tankyrase, Wnt, colorectal cancer, resistance, mTOR

## Abstract

Activation of Wnt/β-catenin signaling is essential for colorectal carcinogenesis. Tankyrase, a member of the poly(ADP-ribose) polymerase (PARP) family, is a positive regulator of the Wnt/β-catenin signaling. Accordingly, tankyrase inhibitors are under preclinical development for colorectal cancer (CRC) therapy. However, Wnt-driven colorectal cancer cells are not equally sensitive to tankyrase inhibitors, and cellular factors that affect tankyrase inhibitor sensitivity remain elusive. Here, we established a tankyrase inhibitor-resistant cell line, 320-IWR, from Wnt/β-catenin-dependent CRC COLO-320DM cells. 320-IWR cells exhibited resistance to tankyrase inhibitors, IWR-1 and G007-LK, but remained sensitive to a PARP-1/2 inhibitor, olaparib, and several anti-CRC agents. In 320-IWR cells, nuclear localization of active β-catenin was decreased and expression of β-catenin target genes was constitutively repressed, suggesting that these cells repressed the Wnt/β-catenin signaling and were dependent on alternative proliferation pathways. 320-IWR cells exhibited upregulated mTOR signaling and were more sensitive to mTOR inhibition than the parental cells. Importantly, mTOR inhibition reversed resistance to tankyrase inhibitors and potentiated their anti-proliferative effects in 320-IWR cells as well as in CRC cell lines in which the mTOR pathway was intrinsically activated. These results indicate that mTOR signaling confers resistance to tankyrase inhibitors in CRC cells and suggest that the combination of tankyrase and mTOR inhibitors would be a useful therapeutic approach for a subset of CRCs.

## INTRODUCTION

Colorectal cancer (CRC) is a leading cause of cancer death worldwide. In the treatment of metastatic CRC, conventional chemotherapy and several molecularly-targeted drugs are currently used as standard drugs [1]. However, the effectiveness of these drugs is limited, and development of additional new drugs is required to improve treatment outcome. In CRC, multi-step genetic changes drive tumor development [2]. Together with mutations in oncogenes and tumor suppressor genes, such as *KRAS*, *p53*, and *SMAD4*, activation of the canonical Wnt/β-catenin signaling pathway is a critical event in colorectal tumorigenesis. The Wnt/β-catenin signaling activation is mainly caused by loss-of-function mutations in the adenomatous polyposis coli (*APC*) gene [3]. In most sporadic CRC cases, mutations in *APC* occur, which lead to stabilization of β-catenin and activation of downstream TCF/LEF-mediated transcription [3, 4]. The Wnt/β-catenin pathway plays an essential role not only in CRC initiation but also in tumor maintenance [5]. These observations indicate that Wnt/β-catenin signaling is a rational therapeutic target for CRC.

Tankyrase is a member of the poly(ADP-ribose) polymerase (PARP) family of proteins, originally identified as a telomeric repeat binding factor-interacting protein [6]. Tankyrase recognizes its substrate proteins through the multiple ankyrin repeat cluster domains for PARylation and is involved in telomere homeostasis and in other biological events such as mitosis [6, 7]. Since the discovery of tankyrase as a positive regulator of Wnt/β-catenin signaling [8], tankyrase has particularly been considered as a promising molecular target for CRC therapy and studies on tankyrase inhibitor development is actively ongoing. In Wnt/β-catenin pathway, tankyrase PARylates Axin, a negative regulator of the Wnt pathway, leading to its ubiquitylation by RNF146 and proteasome-mediated degradation [9]. As a result, tankyrase causes β-catenin stabilization and positively regulates the Wnt/β-catenin signaling pathway. Recently, several tankyrase inhibitors have been developed, including XAV939, IWR-1, G007-LK and AZ1366 [10–13]. In CRC cells, tankyrase inhibitor treatment particularly accumulates Axin2 protein level and causes β-catenin degradation. Among the tankyrase inhibitors reported, G007-LK and AZ1366 were shown to effectively suppress CRC growth *in vivo*, utilizing CRC cell lines and patient-derived tumor xenograft, respectively [12, 13].

However, recent reports have shown that not all Wnt/β-catenin-driven CRC cells are sensitive to tankyrase inhibitors [12, 13]. So far, little information is available as to what determines cellular sensitivity to tankyrase inhibitors. To establish successful tankyrase-targeting therapy for CRC treatment, it is critical to identify the cellular factors that affect sensitivity to the agents and further develop predictive biomarkers for the selection of patient groups to undergo therapy.

To understand the molecular pathways that affect cellular sensitivity to tankyrase inhibitors, we established a novel tankyrase inhibitor-resistant cell line, 320-IWR, from Wnt/β-catenin-dependent CRC COLO-320DM cells. Through the analysis of these resistant cells, we identified the mTOR pathway, an important proliferation factor in various cancers including CRC, as a crucial resistant factor to tankyrase inhibitors.

## RESULTS

### Tankyrase inhibitors suppress CRC COLO-320DM cell proliferation through β-catenin degradation

**Figure 1:**
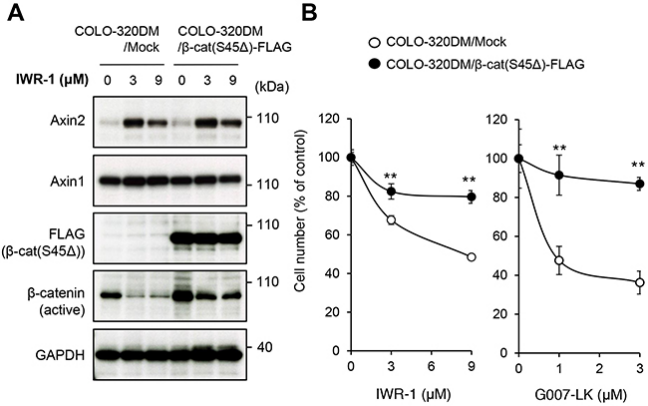
Tankyrase inhibitors suppress colorectal cancer COLO-320DM cell proliferation through inhibition of the β-catenin pathway **(A)** Expression of constitutively active β-catenin in COLO-320DM cells. Cells were transfected with the plasmid vector expressing active β-catenin (Ser45Δ) with a FLAG epitope tag or control vector (Mock) as described in the Materials and Methods. At 24 h after transfection, cells were left untreated or treated with IWR-1 for 16 h. Protein levels of the exogenously expressed constitutively active β-catenin (FLAG), active β-catenin which was dephosphorylated on Ser37 or Thr41 (endogenous and exogenous) and Axin1 and 2 were evaluated by western blot analysis. **(B)** Effect of constitutively active β-catenin expression on tankyrase inhibitor-induced growth inhibition of COLO-320DM cells. COLO-320DM cells transfected with active β-catenin (Ser45Δ) or control vector (Mock) were treated with tankyrase inhibitors, IWR-1 or G007-LK, for 120 h. Cell numbers were evaluated as described in the Materials and Methods. Error bars represent standard deviation (SD) of three independent experiments. Statistical significance was evaluated by Tukey-Kramer test (*: *P* < 0.05; **: *P* < 0.01).

Among the *APC*–mutated CRC cell lines, COLO-320DM cells are sensitive to tankyrase inhibitors [12]. To clarify the significance of β-catenin downregulation after drug treatment in the suppression of COLO-320DM cell proliferation, we overexpressed constitutively active β-catenin with a deleted mutation at Ser45 [β-cat(S45Δ)-FLAG] in the cells. This β-catenin mutant is not phosphorylated by casein kinase I α (CK1α) at Ser45 and is thereby resistant to subsequent phosphorylation at Ser41, Ser37 and Ser33 by glycogen synthase kinase 3β (GSK-3β), which is required for proteasome-mediated degradation [14, 15]. It was also shown that the β-cat(S45Δ) stimulates TCF reporter activity much stronger than the wild-type β-catenin even without Wnt ligand [16]. As expected, when COLO-320DM cells were treated with IWR-1, a tankyrase inhibitor, Axin2 protein accumulated and endogenous active β-catenin protein levels were reduced (Figure 1A). In contrast, β-cat(S45Δ)-FLAG was resistant to degradation even upon the tankyrase inhibitor treatment and active β-catenin levels were maintained after inhibitor treatment (Figure 1A). Importantly, growth inhibition by IWR-1 and another tankyrase inhibitor G007-LK was strongly prevented in these β-catenin mutant-expressing cells (Figure 1B). These results indicate that down-regulation of active β-catenin is a critical step in the growth inhibition of COLO-320DM cells treated with tankyrase inhibitors.

### Establishment of tankyrase inhibitor-resistant 320-IWR cells

**Figure 2:**
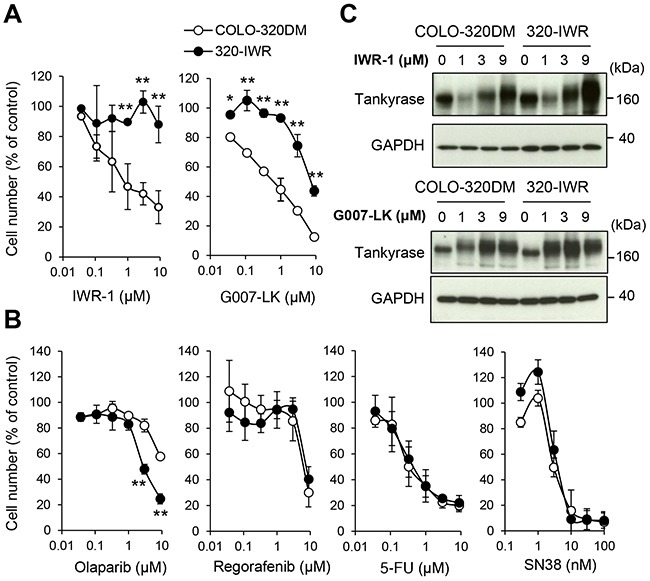
Establishment of 320-IWR, a tankyrase inhibitor-resistant sub-cell line of COLO-320DM cells **(A, B)** Selective resistance of 320-IWR cells to tankyrase inhibitors. COLO-320DM and 320-IWR cells were treated with IWR-1 or G007-LK **(A)** or with olaparib, regorafenib, 5-fluorouracil (5-FU), or SN38, the active metabolite of irinotecan **(B)** for 120 h. Cell numbers were evaluated as in Materials and Methods. Error bars represent standard deviation (SD) of three independent experiments. Statistical significance was evaluated by Tukey-Kramer test (*: *P* < 0.05; **: *P* < 0.01). **(C)** Effect of tankyrase inhibitors on tankyrase protein levels in COLO-320DM and 320-IWR cells. Cells were treated with IWR-1 or G007-LK at the indicated concentrations for 16 h. Protein levels of tankyrase and GAPDH as a loading control were evaluated by western blot analysis.

To understand the mechanism of resistance to tankyrase inhibitors in CRC cells, we established tankyrase inhibitor-resistant cells from COLO-320DM cells. IWR-1 at the concentration of 3 μM induced Axin2 accumulation and subsequent down-regulation of active β-catenin, leading to cell growth inhibition (Figure 1A and 2A). Hence, we continuously treated COLO-320DM cells with IWR-1 at this concentration for 173 days and successfully established a tankyrase inhibitor-resistant cell line, designated as 320-IWR. The morphology of 320-IWR cells was similar to that of the parental COLO-320DM cells (Supplementary Figure 1A). The proliferation rate of 320-IWR cells was almost comparable to that of the parental cells although the resistant cells grew slightly slower (Supplementary Figure 1B): the doubling times of COLO320-DM and 320-IWR cells were 20 h and 22 h, respectively.

320-IWR cells showed marked resistance to IWR-1 (Figure 2A, left). The GI_50_ values of IWR-1 in COLO-320DM and 320-IWR cells were 0.87 μM and > 9 μM, respectively, indicating that 320-IWR cells were more than 10.3-fold resistant to IWR-1. 320-IWR cells also showed cross-resistance to G007-LK, another tankyrase inhibitor with a different chemical structure to IWR-1 (Figure 2A, right). The GI_50_ values of G007-LK in COLO320DM and 320-IWR cells were 0.71 μM and 7.0 μM, respectively, indicating that 320-IWR cells were 9.9-fold resistant to G007-LK. Flow cytometry analysis revealed that tankyrase inhibitors suppressed COLO-320DM cell growth without significant apoptosis induction (as revealed by sub-G1 fraction) or arrest at specific phase of the cell cycle (Supplementary Figure 2A and Supplementary Table 1). In addition, there was no marked difference in cell cycle distribution between COLO-320DM and 320-IWR cells, though slight decrease of G1 and S phase and increase of G2/M phase cells were observed in 320-IWR cells. To examine whether the tankyrase inhibitor-resistant phenotype was stable, we cultured 320-IWR cells for 36 days in the absence of IWR-1. After the drug-free culture, 320-IWR cells maintained resistance to IWR-1 and G007-LK (Supplementary Figure 2B). To confirm that 320-IWR cells were resistant to tankyrase inhibition, we further knocked down the two tankyrase isozymes, tankyrase-1 and -2, simultaneously. We found that 320-IWR cells were highly resistant to tankyrase knockdown (Supplementary Figure 3).

320-IWR cells did not show cross-resistance to olaparib, a PARP1/2 inhibitor, but rather showed collateral sensitivity to the agent (Figure 2B). 320-IWR cells did not show resistance to therapeutic drugs for CRC, including regorafenib, 5-fluorourail (5-FU) or SN38, the active metabolite of irinotecan (Figure 2B). These observations indicate that 320-IWR is a stable cell line, showing selective resistance to tankyrase inhibitors.

### Alteration of canonical Wnt/β-catenin signaling in 320-IWR cells

**Figure 3:**
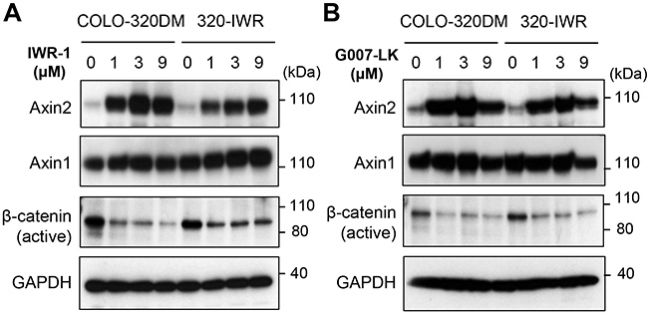
Alteration of canonical Wnt/β-catenin signaling in 320-IWR cells **(A, B)** Effect of tankyrase inhibitors on protein levels of Wnt/β-catenin pathway regulators in COLO-320DM and 320-IWR cells. Cells were treated with IWR-1 and G007-LK at the indicated concentrations for 16 h. Protein levels of Axin1, 2, active β-catenin (dephosphorylated on Ser37 or Thr41) and GAPDH as a loading control were evaluated by western blot analysis.

COLO-320DM and 320-IWR cells expressed comparable levels of tankyrase (Figure 2C). Because tankyrase PARylates itself for proteasome-dependent degradation, tankyrase inhibition leads to accumulation of tankyrase itself [12]. IWR-1 and G007-LK induced accumulation of tankyrase in 320-IWR cells in a similar manner to the parental cells (Figure 2C). These results indicate that both inhibitors were incorporated into the resistant cells and were still effective in suppressing tankyrase PARP activity. We further sequenced two tankyrase isozymes, tankyrase-1 and tankyrase-2, in 320-IWR cells, and no acquired mutation was found in these genes (data not shown). Consistent with these results, IWR-1 and G007-LK caused accumulation of Axin2 and down-regulation of active β-catenin in 320-IWR cells as well as in the parental cells (Figure 3A and 3B). In 320-IWR cells, we observed marginal reduction in Axin2 protein accumulation and β-catenin decrease after tankyrase inhibitor treatment, which could lead to slightly reduced pathway inhibition as a cause of resistance to tankyrase inhibitors. Overall, these data indicate that the tankyrase–Axin2–β-catenin axis in 320-IWR cells was still sensitive to tankyrase inhibitors.

**Figure 4:**
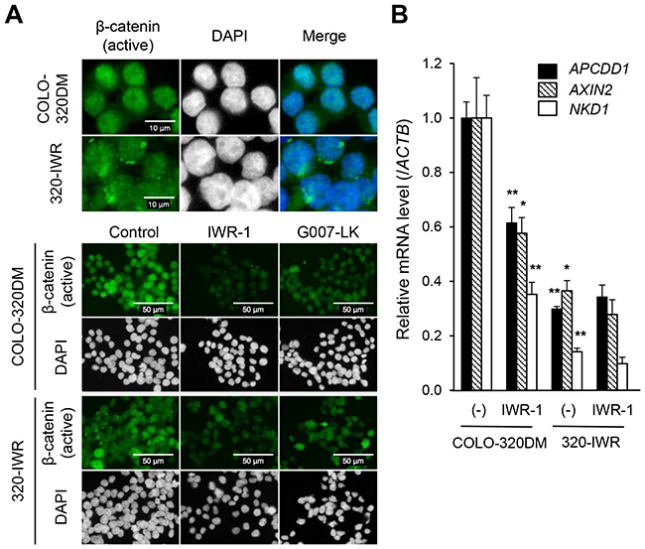
Constitutively repressed Wnt/β-catenin signaling in 320-IWR cells **(A)** Subcellular localization of β-catenin in COLO-320DM and 320-IWR cells. Cells were left untreated (upper panels and control in lower panels) or treated with 3 μM IWR-1 or 3 μM G007-LK for 16 h (lower panels). Cells were subjected to immunofluorescence staining with anti-non-phospho-β-catenin (green). DAPI staining of nuclear DNA is shown in white. **(B)** Expression of genes downstream of the Wnt/β-catenin pathway in COLO-320DM and 320-IWR cells. Cells were left untreated (−) or treated with 3 μM IWR-1 for 16 h. Total RNA was prepared and the expression levels of the Wnt/β-catenin pathway genes were analyzed using RT-qPCR. β-Actin (*ACTB*) expression was analyzed to normalize the data. Error bars represent standard deviation (SD) of three independent experiments. Statistical significance between untreated and IWR-1-treated COLO-320DM cells or between untreated COLO-320DM and 320-IWR cells was evaluated by Student *t* test (*: *P* < 0.05; **: *P* < 0.01).

We next examined the subcellular localization of β-catenin. As shown in Figure 4A, the active β-catenin protein was mainly localized in the nuclei of COLO-320DM cells. Meanwhile, in 320-IWR cells, it was diffusely distributed in both the cytoplasm and the nucleus. Given that the total active β-catenin levels were similar between the two cell lines (Figure 3A and 3B), these results indicate that nuclear active β-catenin levels were decreased in 320-IWR cells.

Consistent with these observations, the expression of Wnt/β-catenin-TCF/LEF pathway genes, *APCDD1*, *AXIN2*, *NKD1*, *MYC*, *LEF1*, and *JAG1* [12, 17] was strongly repressed in 320-IWR cells even in the absence of tankyrase inhibitors (Figure 4B, Supplementary Figure 4A). These genes included *MYC*, an essential factor in Wnt/β-catenin-dependent intestinal tumorigenesis [18]. TCF-dependent transcription activity was also decreased significantly in 320-IWR cells (Supplementary Figure 4B). On the other hand, some β-catenin target genes (*LGR5* and *BIRC5*) were not down-regulated in 320-IWR cells (Supplementary Figure 4A), and the decrease of TCF promoter activity in 320-IWR cells was not so drastic as transcriptional repression of β-catenin target genes (Supplementary Figure 4B). These observations suggest that the transcriptional repression of β-catenin target genes in 320-IWR cells would not only be caused by the decrease in the nuclear β-catenin levels and transcriptional activity, but also by other factors such as promoter methylation of the genes.

To confirm the role of β-catenin pathway in 320-IWR cells further, we knocked down β-catenin by siRNA (Supplementary Figure 5A). 320-IWR cells were significantly more resistant to β-catenin repression than COLO-320DM cells (Supplementary Figure 5B). However, 320-IWR cells were more sensitive to β-catenin knockdown than to the tankyrase inhibitor-induced down-regulation of active β-catenin (Figures 2A and 3). This discrepancy would be due to the differential action of tankyrase inhibitors and β-catenin knockdown: tankyrase inhibitor treatment repressed active β-catenin expression but not markedly total β-catenin level, whereas β-catenin knockdown repressed both the active and total β-catenin levels clearly (Supplementary Figure 5A).

Collectively, these data indicate that 320-IWR cells retained the ability to proliferate despite the repressed Wnt/β-catenin signaling. Our observations suggest that the resistant cells could be less addicted to the Wnt/β-catenin signaling but depend on alternative signaling pathways for their proliferation.

### Activation of the mTOR signaling pathway in 320-IWR cells

We next performed a genome-wide transcriptome analysis. We extracted genes that were up- or down-regulated in 320-IWR cells: genes up- or down-regulated more than 5-fold in 320-IWR cells are shown in Supplementary Table 2. Subsequent gene ontology (GO) analysis revealed that development- or differentiation-related genes were differentially expressed between 320-IWR and the parental COLO-320DM cells (Table 1). Of note, the GO analysis also revealed altered expression of genes related to ‘catenin import into nucleus’, which could be responsible for the altered subcellular localization of active β-catenin protein in 320-IWR cells (Figure 4A). As for the genes involved in drug detoxification or drug transport, we did not observe any increases in gene expression in 320-IWR cells (Supplementary Figure 6), excluding the possibility that 320-IWR cells acquired an enhanced ability to inactivate or efflux the tankyrase inhibitors.

**Table 1:** Gene Ontology (GO) analysis on the genes selectively expressed in 320-IWR cells

	GO number	GO term	p-value		GO number	GO term	p-value
1	GO:0009653	anatomical structure morphogenesis	1.98E-12	22	GO:0045595	regulation of cell differentiation	5.65E-07
2	GO:0060968	regulation of gene silencing	5.06E-12	23	GO:0022008	neurogenesis	5.45E-07
3	GO:0048731	system development	3.51E-11	24	GO:0072001	renal system development	6.51E-07
4	GO:0007275	multicellular organismal development	1.45E-09	25	GO:0061138	morphogenesis of a branching epithelium	6.61E-07
5	GO:0048856	anatomical structure development	1.86E-09	26	GO:0035108	limb morphogenesis	7.80E-07
6	GO:0048513	organ development	1.40E-08	27	GO:0035107	appendage morphogenesis	7.80E-07
7	GO:0044707	single-multicellular organism process	2.28E-08	28	GO:0001822	kidney development	7.30E-07
8	GO:0030154	cell differentiation	2.58E-08	29	GO:0048754	branching morphogenesis of an epithelial tube	8.67E-07
9	GO:0002009	morphogenesis of an epithelium	2.94E-08	30	GO:0030111	regulation of Wnt signaling pathway	1.05E-06
10	GO:0032501	multicellular organismal process	4.64E-08	31	GO:0035295	tube development	1.04E-06
11	GO:0009887	organ morphogenesis	5.63E-08	32	GO:0048468	cell development	1.14E-06
12	GO:0032502	developmental process	5.95E-08	33	GO:0061312	BMP signaling pathway involved in heart development	1.31E-06
13	GO:0048869	cellular developmental process	7.13E-08	34	GO:0050793	regulation of developmental process	1.27E-06
14	GO:0048729	tissue morphogenesis	7.71E-08	35	GO:0045597	positive regulation of cell differentiation	1.36E-06
15	GO:0044767	single-organism developmental process	1.48E-07	36	GO:0030182	neuron differentiation	1.52E-06
16	GO:0060429	epithelium development	2.76E-07	37	GO:0001763	morphogenesis of a branching structure	1.54E-06
17	GO:0007399	nervous system development	3.28E-07	38	GO:0060173	limb development	2.37E-06
18	GO:0035239	tube morphogenesis	3.41E-07	39	GO:0048736	appendage development	2.37E-06
19	GO:0060562	epithelial tube morphogenesis	3.81E-07	40	GO:0001655	urogenital system development	2.86E-06
20	GO:0048699	generation of neurons	3.76E-07	41	GO:0003002	regionalization	3.33E-06
21	GO:2000738	positive regulation of stem cell differentiation	4.50E-07	42	GO:0009954	proximal/distal pattern formation	3.48E-06
43	GO:2000736	regulation of stem cell differentiation	3.60E-06	57	GO:0051094	positive regulation of developmental process	9.35E-06
44	GO:0048598	embryonic morphogenesis	4.20E-06	58	GO:0072006	nephron development	1.13E-05
45	GO:0030326	embryonic limb morphogenesis	5.66E-06	59	GO:0002062	chondrocyte differentiation	1.14E-05
46	GO:0035113	embryonic appendage morphogenesis	5.66E-06	60	GO:0035412	regulation of catenin import into nucleus	1.29E-05
47	GO:0022603	regulation of anatomical structure morphogenesis	6.13E-06	61	GO:0090092	regulation of transmembrane receptor protein Ser/Thr kinase signaling pathway	1.39E-05
48	GO:0060828	regulation of canonical Wnt signaling pathway	7.02E-06	62	GO:0003307	regulation of Wnt signaling pathway involved in heart development	1.43E-05
49	GO:0003129	heart induction	8.79E-06	63	GO:0000904	cell morphogenesis involved in differentiation	1.46E-05
50	GO:0003130	BMP signaling pathway involved in heart induction	8.79E-06	64	GO:0010717	regulation of epithelial to mesenchymal transition	1.74E-05
51	GO:0003133	endodermal-mesodermal cell signaling	8.79E-06	65	GO:0090090	negative regulation of canonical Wnt signaling pathway	1.89E-05
52	GO:0003134	endodermal-mesodermal cell signaling involved in heart induction	8.79E-06	66	GO:0051239	regulation of multicellular organismal process	2.23E-05
53	GO:2000026	regulation of multicellular organismal development	8.03E-06	67	GO:0009611	response to wounding	2.30E-05
54	GO:0090081	regulation of heart induction by regulation of canonical Wnt signaling pathway	8.79E-06	68	GO:0051093	negative regulation of developmental process	2.39E-05
55	GO:1901213	regulation of transcription from RNA pol II promoter involved in heart development	9.09E-06	69	GO:0035413	positive regulation of catenin import into nucleus	2.54E-05
56	GO:0072073	kidney epithelium development	9.45E-06	70	GO:0030178	negative regulation of Wnt signaling pathway	2.53E-05

NOTE: cDNA microarray analysis was performed on COLO-320DM and 320-IWR cells using the GeneChip Human Genome U133 Plus 2.0 array (Affymetrix). Gene ontology (GO) analysis was done with the GeneSpring GX software (Agilent Technologies) on the genes that were more than 2-fold up- or down-regulated in 320-IWR cells.

**Figure 5:**
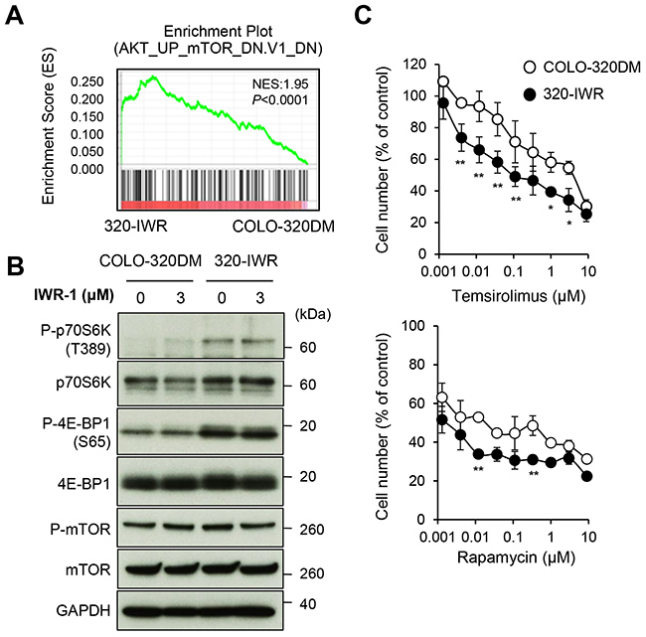
Activation of mTOR signaling pathway in tankyrase inhibitor-resistant 320-IWR cells **(A)** Representative result of the Gene Set Enrichment Analysis (GSEA) showing enrichment of the AKT–mTOR pathway-related gene signature in 320-IWR cells. **(B)** Elevated phosphorylation of mTOR pathway regulators in 320-IWR cells. Cells were left untreated or treated with 3 μM IWR-1 for 16 h. Protein levels and phosphorylation status of mTOR pathway regulators were evaluated by western blot analysis. **(C)** Preferential sensitivity of 320-IWR cells to mTOR inhibitors. COLO-320DM and 320-IWR cells were treated with mTOR inhibitors, temsirolimus or rapamycin, for 120 h. Cell numbers were evaluated as in the Materials and Methods. Error bars represent standard deviation (SD) of three independent experiments. Statistical significance was evaluated by Tukey-Kramer test (*: *P* < 0.05; **: *P* < 0.01).

Gene set enrichment analysis (GSEA) [19] further revealed that the gene signature related to the mTOR pathway was significantly enriched in 320-IWR cells (Figure 5A). The mTOR pathway is often activated in various cancers including CRC and plays a critical role in tumor growth [20]. Phosphorylation of p70S6 kinase (p70S6K) and 4E-BP1, the downstream signaling molecules of the mTOR pathway, was clearly elevated in 320-IWR cells, whereas the phosphorylation levels of mTOR itself were similar between 320-IWR and COLO-320DM cells (Figure 5B). Importantly, the mTOR inhibitors temsirolimus and rapamycin suppressed p70S6K phosphorylation (Supplementary Figure 7A) and showed more prominent anti-proliferative effects on 320-IWR cells when compared with the parental cells (Figure 5C). These results indicate that the activated mTOR pathway could serve as a selective proliferation factor in 320-IWR cells. On the other hand, we could not exclude the possibility of additional alteration in other signaling pathway in 320-IWR cells, because the differential sensitivity to the mTOR inhibitors between 320-IWR and the parental cells was not dramatic.

### Activation of mTOR pathway causes resistance to tankyrase inhibitors

**Figure 6:**
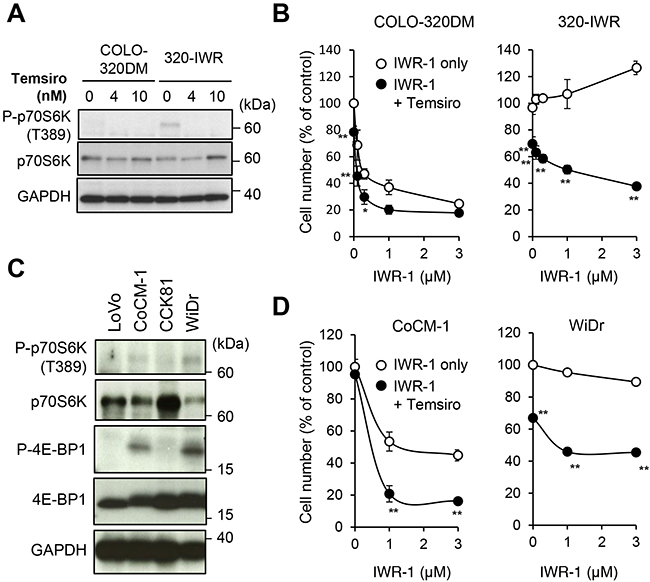
mTOR pathway activation confers resistance to tankyrase inhibition in 320-IWR cells **(A)** Inhibition of p70S6 kinase (p70S6K) phosphorylation by an mTOR inhibitor. Cells were treated with temsirolimus (Temsiro) at the indicated concentrations for 2 h. Protein levels and phosphorylation status of p70S6K were evaluated by western blot analysis. **(B)** Reversal of tankyrase inhibitor resistance in 320-IWR cells by temsirolimus. COLO-320DM and 320-IWR cells were treated with IWR-1 and 4 nM temsirolimus (Temsiro) together at the indicated concentrations for 120 h. Cell numbers were calculated as in the Materials and Methods. Error bars represent standard deviation (SD) of three independent experiments. Statistical significance was evaluated by Tukey-Kramer test (*:*P* < 0.05; **: *P* < 0.01). **(C)** Expression and phosphorylation of p70S6 kinase (p70S6K) and 4E-BP1 in CRC cells. Protein levels and phosphorylation of p70S6K were evaluated by western blot analysis. **(D)** Potentiation of the anti-proliferative effect of IWR-1 by temsirolimus. Cells were treated with IWR-1 at the indicated concentrations and 10 nM temsirolimus (Temsiro) together for 120 h. Cell numbers were calculated. Error bars represent standard deviation (SD) of three independent experiments. Statistical significance was evaluated by Tukey-Kramer test (**: *P* < 0.01).

To examine the role of mTOR signaling in resistance to tankyrase inhibitors, we co-treated 320-IWR and parental cells with tankyrase inhibitors and mTOR inhibitor temsirolimus. As shown in Figure 6A, a sub-lethal dose (4 nM) of temsirolimus suppressed p70S6K phosphorylation and dramatically reversed the resistance of 320-IWR cells to IWR-1 (Figure 6B) and G007-LK (Supplementary Figure 7B). Isobologram analysis [21] revealed that the combinational effect of tankyrase inhibitor and mTOR inhibitor in 320-IWR cells was synergistic (Supplementary Figure 7C). To confirm these observations, we further obtained multiple cell clones from 320-IWR cells. In these clones, mTOR activation, as revealed by p70S6K phosphorylation, was observed and temsirolimus enhanced the growth suppressive effect of IWR-1 (Supplementary Figure 8). At the same time, we also obtained a clone with less mTOR activation (#3) in which temsirolimus was less effective. These observations suggest additional, mTOR-independent mechanisms of resistance to tankyrase inhibitors in the clone.

The mTOR pathway is frequently activated in CRC cells [20]. To examine whether mTOR signaling could also be involved in intrinsic resistance of CRC cells to tankyrase inhibitors, we analyzed the mTOR pathway in CRC cell lines. Among the cell lines, we observed phosphorylated p70S6K and 4E-BP1 in CoCM-1 and WiDr cells (Figure 6C). Importantly, temsirolimus enhanced IWR-1-induced growth suppression in these cells (Figure 6D). Isobologram analysis confirmed that the combinational effect of tankyrase inhibitor and mTOR inhibitor in these cells was synergistic (Supplementary Figure 7C). We also tested the combinational effect of tankyrase/mTOR inhibition in two additional CRC cell lines, HCC2998 and DLD-1, which have KRAS mutations and more common APC mutations with two 20-AAR (20-amino acid repeat) motifs. Temsirolimus inhibited the growth of DLD-1 but not HCC2998, and gave no synergistic effect with IWR-1 in these cells (Supplementary Figure 7D). Collectively, these observations indicate that the mTOR pathway is a critical resistant factor to tankyrase inhibitors in a subset of CRC cells.

## DISCUSSION

In this study, we established a tankyrase inhibitor-resistant cell line, 320-IWR. 320-IWR cells were resistant to multiple tankyrase inhibitors with different structures, whereas they did not show cross-resistance to a PARP-1/2 inhibitor. These data indicate that the cells were specifically resistant to tankyrase inhibitors but not widely to PARP inhibition. Tankyrase inhibitors are classified into two types: one type targeting the nicotinamide subsite of the tankyrase protein, which is conserved in various PARPs, and the other targeting a unique adenosine subsite that is more potent and specific to tankyrase [22]. Since IWR-1 and G007-LK belong to the latter class of tankyrase inhibitors, it is reasonable that the resistant mechanisms are also specific to tankyrase inhibitors. Acquired resistance to molecularly -targeted drugs is often caused by mutations around the drug-binding sites in target molecules [23]. However, no acquired mutation was found in tankyrase genes in 320-IWR cells, and inhibition of tankyrase PARP activity by the inhibitors was observed in 320-IWR cells in a similar manner to that in the parental COLO-320DM cells, as revealed by accumulation of Axin2 and tankyrase itself, the substrate proteins of tankyrase (Figures 2C and 3A).

320-IWR cells showed collateral sensitivity to a PARP-1/2 inhibitor, olaparib (Figure 2B). These data could provide an important clue to a therapeutic approach for recurrent tumors after treatment with tankyrase inhibitors. PARP-1 and 2 play essentials role in DNA repair, and synthetic lethality is shown between PARP-1/2 inhibition and BRCA1/2 deficiency, essential factors for DNA homologous recombination [24]. Synthetic lethality between tankyrase inhibition and BRCA deficiency was also reported [25]. These observations suggest that tankyrase could also be involved in a particular DNA repair pathway, and alteration of the pathway after long exposure to a tankyrase inhibitor would affect the sensitivity of 320-IWR cells to olaparib.

Our data have shown the involvement of mTOR signaling in resistance to tankyrase inhibitors. At present, it is still elusive as to how the mTOR pathway is activated in the resistant cells. It was reported that PTEN is a substrate of tankyrase, and PARylation of PTEN by tankyrase promotes PTEN degradation and activation of downstream AKT [26]. However, after tankyrase inhibitor treatment, we did not observe any marked changes in the phosphorylation of AKT (Y. Muramatsu, unpublished observation) and its downstream molecule mTOR (Figure 5B) in 320-IWR and COLO-320DM cells. Moreover, in 320-IWR cells, phosphorylation of p70S6K and 4E-BP1, the downstream mediators of mTOR signaling, were elevated without any change in mTOR phosphorylation (Figure 5B). These data indicate that the phosphorylation of p70S6K and 4E-BP1 may not be caused by activation of the AKT–mTOR axis. In the mTOR pathway, there are two regulator complexes, mTORC1 and mTORC2. p70S6K and 4E-BP1 are regulated by the mTORC1 complex, which consists of mTOR and its regulators such as Raptor [20]. Further analyses are required to determine whether components of the mTORC1 complex could be altered in 320-IWR cells. On the other hand, mTOR activation in 320-IWR might be caused by a more dramatic phenotypic change, since continuous treatment with tankyrase inhibitors could affect the Wnt/β-catenin pathway, which is involved in cellular differentiation. Indeed, expression of differentiation-related genes was largely changed between 320-IWR and COLO-320DM cells (Table 1). Particularly, 320-IWR cells overexpressed prominin 1 (CD133) (Supplementary Table 2), a well-known marker of cancer stem cells [27], and mTOR activation was also reported in cancer stem cells [28]. Additional studies would clarify the relationship of tankyrase inhibitor sensitivity to cancer stemness.

Activation of the mTOR pathway emerged as a mechanism of resistance to Wnt/β-catenin pathway inhibition by tankyrase inhibitors. This implies potentially compensatory roles of the two signaling pathways. Indeed, β-catenin was reported to confer resistance to inhibitors of phosphatidylinositol 3-kinase (PI3K) and AKT, upstream regulators of the mTOR pathway [29]. The mTOR pathway is often activated in CRC cells [21], and therefore mTOR activation could not only be involved in the acquired resistance but also in the intrinsic resistance to tankyrase inhibitors in CRC. We identified mTOR signaling-mediated resistance to tankyrase inhibitors in COLO-320DM cells, which possess short truncated APC lacking all the 20-aa repeats [12] and are devoid of KRAS and PIK3CA mutation. In our recent analysis with CRC patient-derived cells, hemizygous short APC mutations were observed in six out of 16 APC-mutated CRCs [30]. According to the TCGA mutational analysis [31], there is also a subclass of CRCs without any mutations in KRAS or PIK3CA. In our data, mTOR inhibitor enhanced tankyrase inhibitor cytotoxicity in CoCM1 cells with PIK3CA(R1023Q) mutation [32], and in WiDr cells having BRAF(V600E) and PIK3CA(P449T) mutation (Figure 6D) [33]. From these results, mTOR pathway would interfere with the anti-proliferative effect of tankyrase inhibitors in several types of CRC. Nevertheless, further validation studies with more comprehensive setting, including larger number of cell lines and patient-derived cells, are needed to evaluate how generalizable the effect would be. Additionally, the combinational effect of tankyrase and mTOR inhibitors should be validated *in vivo*. Our analyses with 320-IWR-derived clones also suggested mTOR-independent mechanisms of tankyrase inhibitor-resistance. Further studies are required to identify these mechanisms of resistance to tankyrase inhibitors.

## MATERIALS AND METHODS

### Cell lines and chemical compounds

Human colorectal cancer COLO-320DM cells were obtained from the American Type Cell Collection (ATCC). Human colorectal cancer LoVo, CoCM-1, CCK-81 and WiDr cells were obtained from the JCRB cell bank (Osaka, Japan). Human colorectal cancer HCC-2998 and DLD-1 cells were obtained as described previously [30]. These cell lines were authenticated by short tandem repeat (STR) analysis (BEX, Tokyo, Japan). STR analysis was also performed in 320-IWR cells and we confirmed that the cells were derived from COLO-320DM cells. Cell culture conditions for each cell line are described in the Supplementary Materials and Methods. Information on chemical compounds used in this study (tankyrase inhibitors, mTOR inhibitors and other agents) is detailed in Supplementary Materials and Methods.

### Vector construction and transfection

Full-length cDNA for constitutively active human β-catenin with a deleted mutation at Ser45 [14] was amplified by PCR using cDNA extracted from human CRC HCT116 cells as a template. The cDNA was cloned into a pLPCX vector (Takara) with a FLAG epitope tag at the carboxyl-terminus to generate pLPCX-β-catenin (Ser45Δ)-FLAG. COLO-320DM cells were transiently transfected with the pLPCX-β-catenin (Ser45Δ)-FLAG or pLPCX control vectors by lipofection with Lipofectamine 2000 reagent (Thermo Fisher Scientific) or by electroporation using the Neon transfection system (Thermo Fisher Scientific) according to manufacturer's instructions.

### Cell proliferation assay

Cell proliferation was evaluated using thiazolyl blue tetrazolium bromide (MTT) (Sigma). Cells were seeded in 96-well microplates and were treated with drugs for 5 days. The detailed procedure is described in Supplementary Materials and Methods.

### Western blot analysis

Cells were lysed in whole cell extract (WCE) lysis buffer [150 mM NaCl, 1.0% Nonidet P-40 (NP-40), 50 mM Tris-HCl, pH 8.0] supplemented with 1× protease inhibitor cocktail (Nacalai) and PhosSTOP phosphatase inhibitor cocktail (Roche). Western blot analysis was performed as described previously [34]. Antibodies used in this study are described in Supplementary Materials and Methods.

### Immunofluorescence staining

Cells were fixed with 2% paraformaldehyde in phosphate-buffered saline (PBS) and were permeabilized with 0.5% NP-40 in PBS. The fixed cells were blocked in PBS containing 1% bovine serum albumin (BSA) and reacted with the rabbit anti-non-phospho (active) β-catenin (S33/S37/T41) antibody (Cell Signaling Technology). This primary antibody was detected using the Alexa 488-conjugated anti-rabbit immunoglobulin (IgG). DNA was stained with 0.2 μg/mL 4′,6-diamidino-2-phenylindole (DAPI).

### Reverse transcription-quantitative PCR (RT-qPCR)

Total RNA was extracted using the RNeasy Mini kit (Qiagen). cDNA was synthesized using SuperScript III First-Strand Synthesis SuperMix for RT-qPCR (Thermo Fisher Scientific). RT-qPCR was performed using LightCycler 480 Probes Master (Roche) and detected using a LightCycler 96 (Roche). A Universal ProbeLibrary Human ACTB Gene Assay was used to detect a reference gene to normalize for differences in the amount of RNA in each sample. All probes were purchased from Roche. Primers and probes for RT-qPCR are shown in the Supplementary Table 3.

### cDNA microarray analysis and Gene Set Enrichment Analysis (GSEA)

Total RNA was extracted as described above. cDNA microarray analysis was performed with the RNAs as described previously [26], using the GeneChip Human Genome U133 Plus 2.0 Array (Affymetrix). Normalization of data and Gene Ontology (GO) analysis were performed with the GeneSpring GX software (Agilent Technologies). Gene Set Enrichment Analysis (GSEA) (Broad Institute) was performed on the website (http://software.broadinstitute.org/gsea/index.jsp).

The gene expression data have been deposited in Gene Expression Omnibus (GEO) and are accessible through the accession number GSE86061. The data were released on November 1, 2016.

## SUPPLEMENTARY MATERIALS FIGURES AND TABLES




