# Supplementary material for: mTOR signaling mediates resistance to tankyrase inhibitors in Wnt-driven colorectal cancer
Source: Oncotarget. 2017 May 24;8(29):47902–15. doi: 10.18632/oncotarget.18146 (PMC5564614; doi:10.18632/oncotarget.18146)
Supplement: Supplementary file 1 [file oncotarget-08-47902-s001.pdf]

mTOR signaling mediates resistance to tankyrase inhibitors in Wnt-driven colorectal cancer

SUPPLEMENTARY MATERIALS AND METHODS

Cell culture conditions

COLO-320DM, 320-IWR, HCC-2998 and DLD-1 cells were cultured in RPMI 1640 medium supplemented with 10% heat-inactivated fetal bovine serum (FBS) and 100 μg/mL kanamycin. LoVo cells were cultured in F12 medium supplemented with 20% heat-inactivated FBS and 100 μg/mL kanamycin. CoCM-1 cells were cultured in RPMI/F12 (1:1) medium supplemented with 10% heat-inactivated FBS and 100 μg/mL kanamycin. CCK81 cells were cultured in EMEM medium supplemented with 10% heat-inactivated FBS and 100 μg/mL kanamycin. WiDr cells were cultured in DMEM medium supplemented with 10% heat-inactivated FBS and 100 μg/mL kanamycin.

Chemical compounds

A tankyrase inhibitor, IWR-1 [10], was purchased from Santa Cruz Biotechnology (Santa Cruz, CA, USA). Another tankyrase inhibitor, G007-LK [12], was kindly provided by Dr. Fumiyuki Shirai (RIKEN). Olaparib, regorafenib and rapamycin were purchased from Selleckchem (Houston, TX, USA). 5-fluorouracil (5-FU), SN38 and temsirolimus were purchased from Sigma (St Louis, MO, USA).

MTT cell proliferation assay

For the measurement of cell viability, thiazolyl blue tetrazolium bromide (MTT) was added to the medium at a final concentration of 0.8 mg/ml and, after incubation for 4 h, the medium containing MTT was removed and dimethyl sulfoxide was then added. Optical density (OD) at 570 nm and 630 nm (reference) were measured using an xMark microplate spectrophotometer (Bio-RAD).

Antibodies for western blot analysis

We used the following antibodies for western blot analysis: anti-tankyrase-1/2 (0.4 μg/ml, sc-8337; Santa Cruz Biotechnology), anti-Axin1 (1:500, 2087; Cell Signaling Technology), anti-Axin2 (1:1,000, #2151; Cell Signaling Technology), anti-phospho-p70S6 kinase (p70S6K) (T389) (1:1,000, #9234; Cell Signaling Technology), anti-p70S6K (1:1,000, #9202; Cell Signaling Technology), anti-phospho-4E-BP1 (S65) (1:1,000, #2855; Cell Signaling Technology), anti-4E-BP1 (1:1,000, #9644; Cell Signaling Technology), anti-phospho-mTOR (S2448) (1:1,000, #5536; Cell Signaling Technology), anti-mTOR (1:1,000, #2983; Cell Signaling Technology), anti-active β-catenin which was dephosphorylated on Ser37 or Thr41 (2 μg/ml, 05-665; MILLIPORE), anti-total β-catenin (1:1,000, #9582S; Cell Signaling Technology), and anti-GAPDH (0.05 μg/ml, 10R-G109a; Fitzgerald). As for the tankyrase-1/2 antibody, our unpublished analysis using tankyrase-1 or tankyrase-2 specific siRNAs revealed that this antibody mainly detects tankyrase-1 in COLO-320DM cells.

Cell cycle and apoptosis assay

Cell cycle change and apoptosis induction was determined by flow cytometry as described previously [35]. Briefly, after the treatment with tankyrase inhibitors, cells were fixed in 70% ethanol. After treatment with ribonuclease A, cells were stained with propidium iodide solution. Cell cycle distribution and apoptotic sub-G1 fraction of the cells were analyzed with a FACSCalibur flow cytometer (Becton Dickinson).

siRNA treatment

Silencer Select siRNAs to tankyrase-1 (s500462, CCACUUGAUUGUUUCUUUAtt), tankyrase-2 [(s37280 (GAGUAAAGAUUGUACAGCUtt) and s37281 (GGAC-AUAGGCACAAACUAAtt)] and negative control siRNAs were purchased from Thermo Fisher Scientific. Tankyrase-1 and -2 siRNA cocktail #A is 1:1 mixture of s500462 and s37280. Tankyrase-1 and -2 siRNA cocktail #B is 1:1 mixture of s500462 and s37281. ON-TARGETplus siRNA SMART pool to human β-catenin gene (CTNNB1) and non-targeting control siRNA were purchased from GE Healthcare Dharmacon. These siRNAs were introduced into cells using Lipofectamine RNAiMAX Transfection Reagent (Thermo Fisher Scientific).

Sequencing of tankyrase genes

Total RNA was extracted and cDNA was synthesized as described in the RT-qPCR section of Materials and Methods. Protein coding regions of tankyrase-1 and -2 genes were amplified by PCR from the synthesized cDNA using the KOD Plus Neo kit (Toyobo Co., Ltd., Osaka, Japan) and were directly sequenced with 3130 Genetic Analyzer (Applied Biosystems).

TCF reporter assay

Reporter assay was done as described previously [30]. Cells were transfected with the firefly luciferase vector with Tcf promoter (pTcf7wt-luc, provided by Dr. Kunitada Shimotohno, National Center for Global Health and Medicine, via RIKEN BioResource Center, Ibaraki, Japan) and the Renilla luciferase vector phRL-CMV (Promega) by using Lipofectamine 2000 reagent (Thermo Fisher Scientific). Relative activity of firefly luciferase to Renilla luciferase was determined with the Dual-Glo Luciferase Assay System (Promega).

Cloning of 320-IWR cells

320-IWR cell-derived clones were obtained by limiting dilution method, seeding cells at the density of 1 cell/100 μL/well in 96-well plates. After two weeks, we obtained multiple single colonies from the plates, and expanded the clones for further analysis.

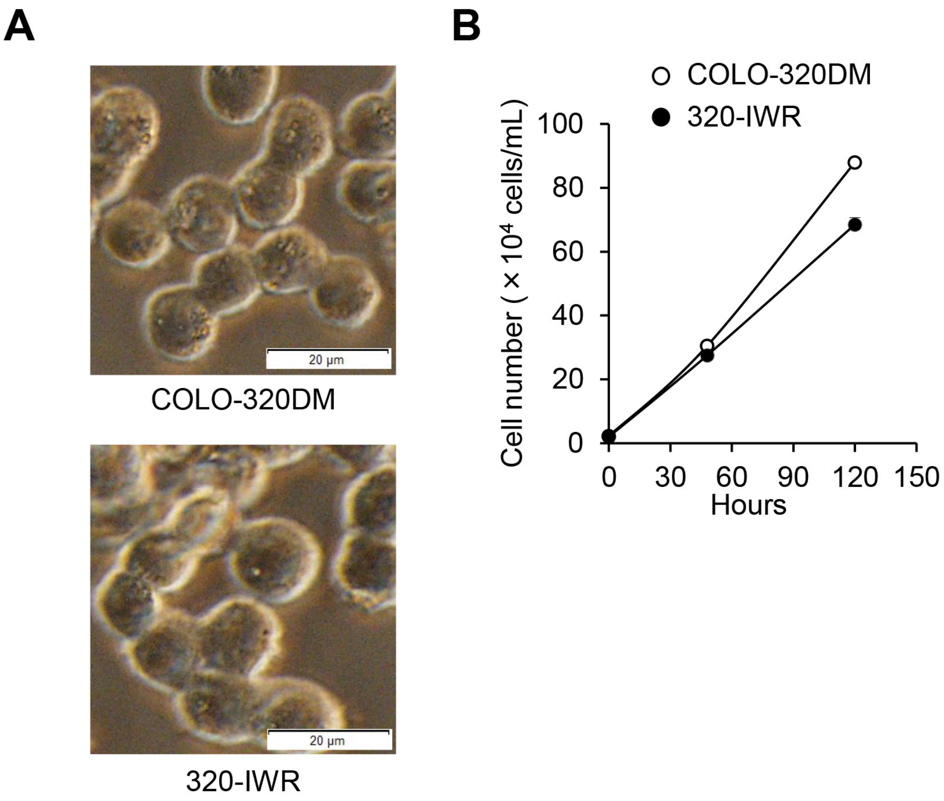

Supplementary Figure 1: Morphology and proliferation rate of 320-IWR cells. (A) Morphology of COLO-320DM and 320-IWR cells. **(B)** Proliferation rate of COLO-320DM and 320-IWR cells. Cell numbers were evaluated as in Materials and Methods. Error bars represent standard deviation (SD) of three independent experiments.

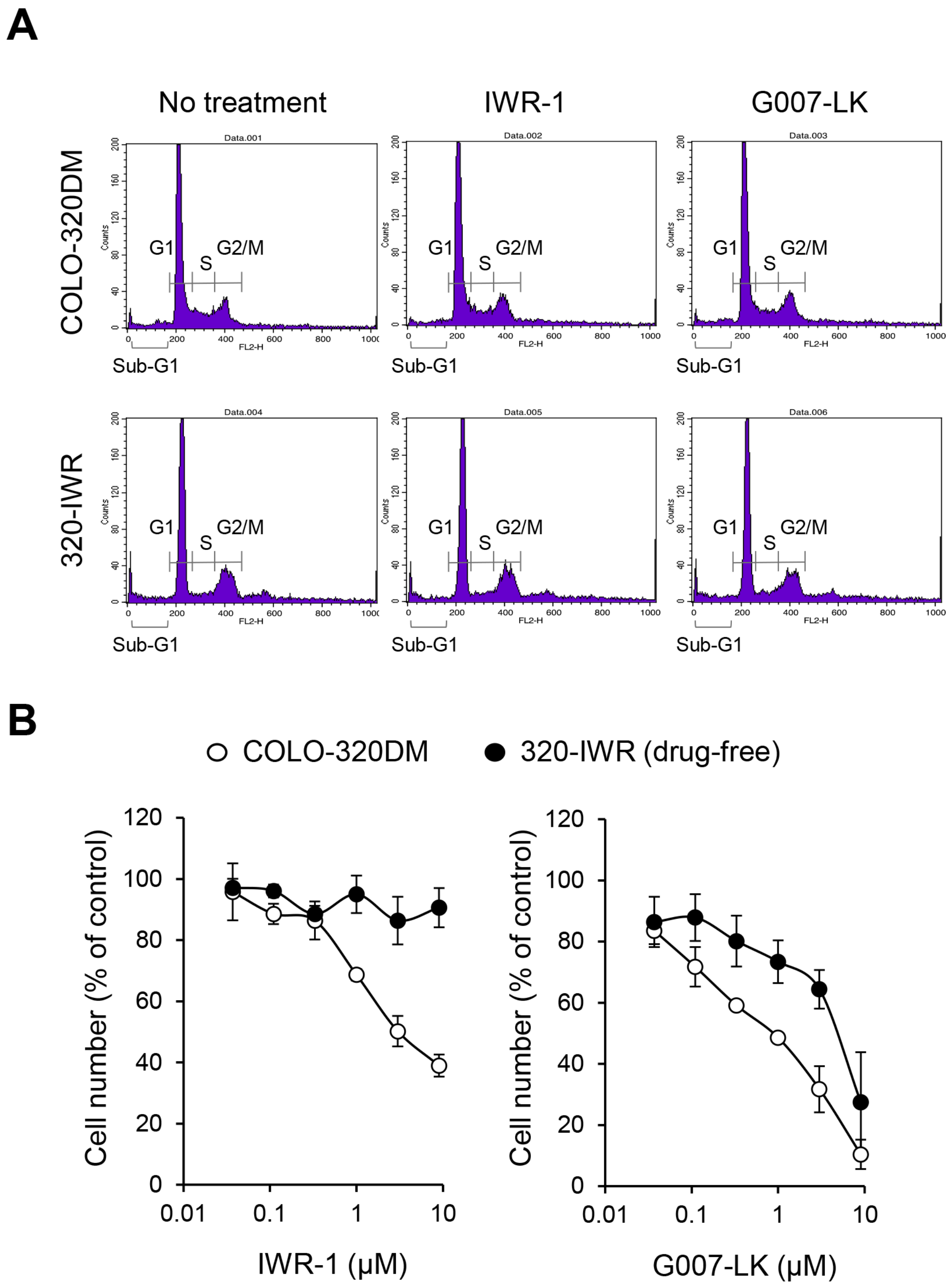

Supplementary Figure 2: Cell cycle distribution and stability of tankyrase inhibitor-resistant phenotype of 320-IWR cells. **(A)** COLO-320DM and 320-IWR cells were treated with tankyrase inhibitors, IWR-1 (9 μM) or G007-LK (1 μM) for 120 h. Cell cycle distribution and apoptotic cells (as revealed by sub-G1 cells) were examined by FACSCalibur flow cytometer as described in Supplementary Materials and Methods. **(B)** 320-IWR cells were cultured for 36 days in the absence of IWR-1. After the long-term drug-free culture, the resulting cells [320-IWR (drug-free)] as well as the parental COLO-320DM cells were treated with IWR-1 or G007-LK for 120 h. Cell numbers were evaluated as in Materials and Methods. Error bars represent standard deviation (SD) of three independent experiments.

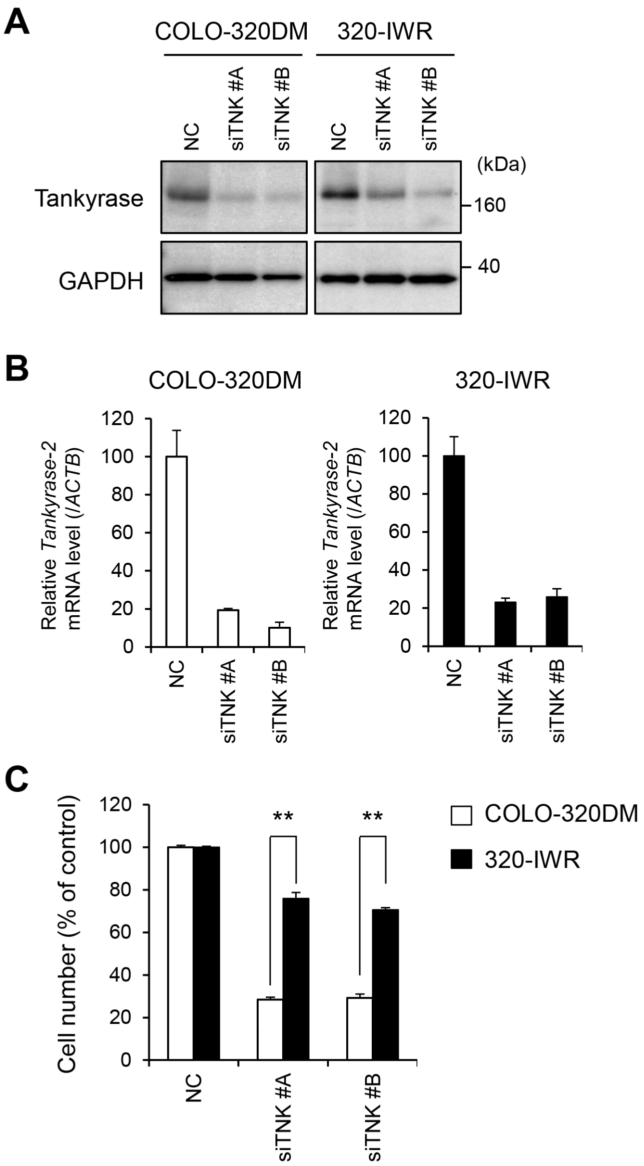

Supplementary Figure 3: Resistance of 320-IWR cells to tankyrase knockdown. Non-silencing control siRNA (NC), cocktail #A of siRNAs to tankyrase-1 and -2 (siTNK #A) or cocktail #B of siRNAs to tankyrase-1 and -2 (siTNK #B) were prepared and introduced into the indicated cells as described in Supplementary Materials and Methods. **(A)** Forty-eight hours after siRNA introduction, cell lysates were prepared and protein levels of tankyrase and GAPDH (as a loading control) were evaluated by western blot analysis. For tankyrase detection, we used anti-tankyrase-1/2 antibody (sc-8337, Santa Cruz Biotechnology), which mainly detects tankyrase-1 in COLO-320DM cells (our unpublished analysis). **(B)** Forty-eight hours after siRNA introduction, total RNA was prepared and *TNKS2* (*tankyrase-2*) expression levels were analyzed using RT-qPCR. β-Actin (*ACTB*) expression was analyzed to normalize the data. Error bars represent standard deviation (SD). **(C)** One-hundred twenty hours after siRNA introduction, cell number was evaluated as in Materials and Methods. Error bars represent standard deviation (SD) of three independent experiments. Statistical significance in the difference between COLO-320DM and 320-IWR cells was evaluated by Student *t* test (**: $P < 0.01$).

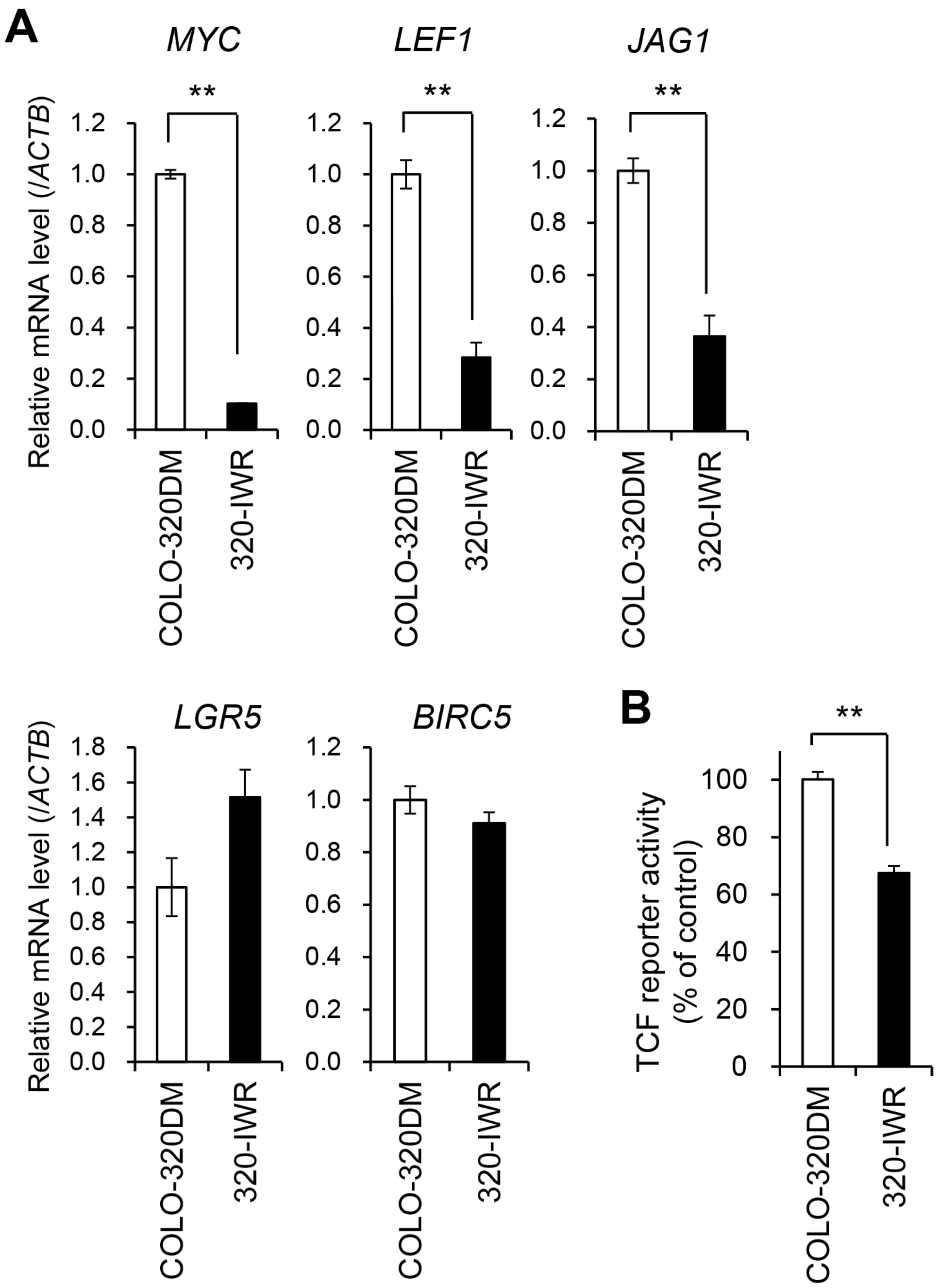

Supplementary Figure 4: Downregulation of Wnt/β-catenin target gene expression and TCF promoter-mediated transcription in 320-IWR cells. (A) Basal level expression of β-catenin target genes in COLO-320DM and 320-IWR cells. Total RNA was prepared and the expression levels of the β-catenin target genes were analyzed using RT-qPCR. β-Actin (*ACTB*) expression was analyzed to normalize the data. Error bars represent standard deviation (SD). For *MYC*, *LEF1* and *JAG1* data, statistical evaluation was done by Student *t* test (**: $P < 0.01$). **(B)** COLO-320DM and 320-IWR cells were transiently transfected with the reporter plasmids, and the Tcf promoter activity normalized by CMV promoter activity (as a control) was determined. Error bars represent standard deviation (SD) of three independent experiments. Statistical evaluation was done by Student *t* test (**: $P < .01$).

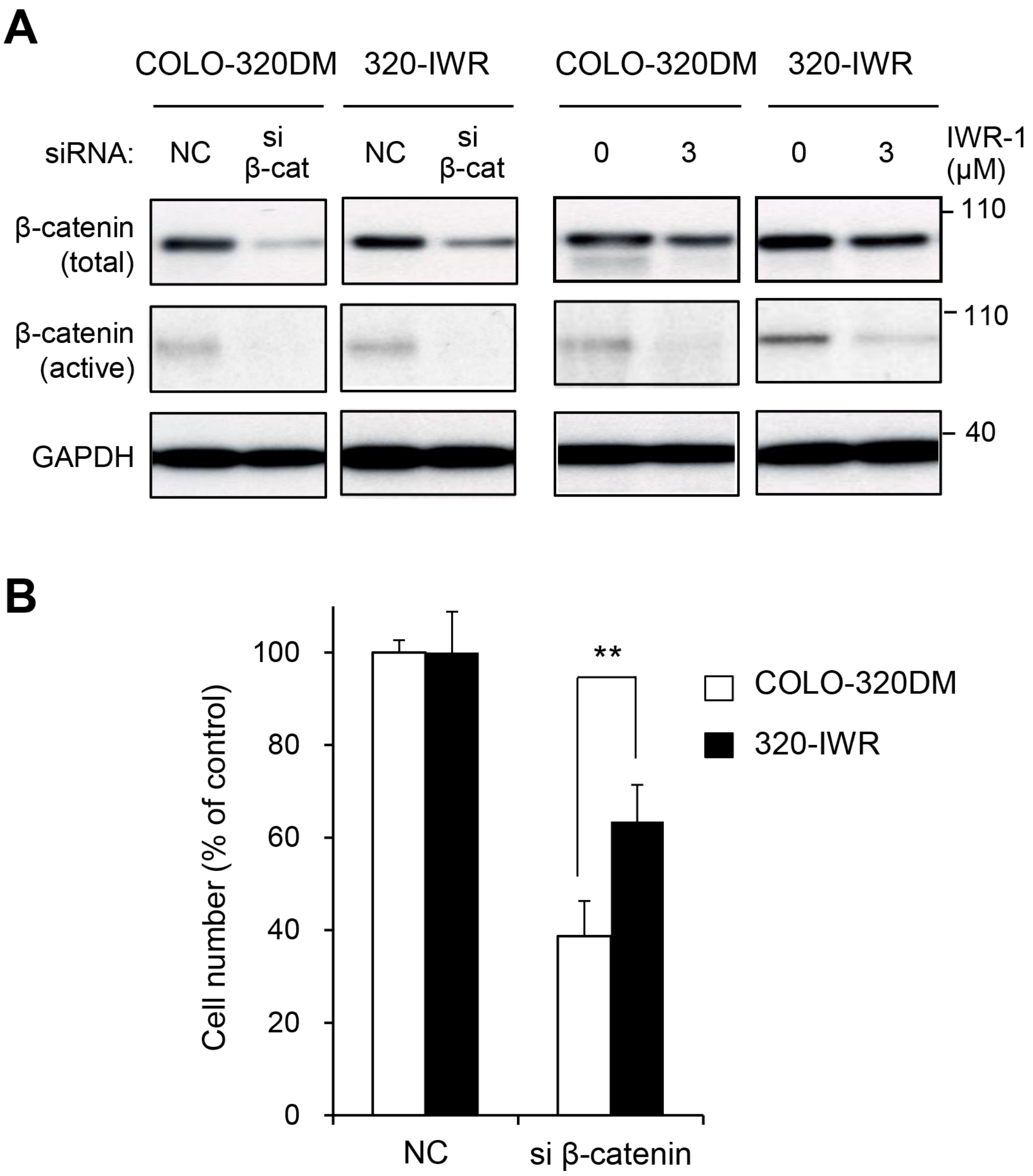

Supplementary Figure 5: Resistance of 320-IWR cells to β-catenin knockdown. Non-silencing control siRNA (NC) or β-catenin siRNA (si β-cat) were introduced into the indicated cells as described in Supplementary Materials and Methods. **(A)** Forty-eight hours after siRNA introduction, cell lysates were prepared and protein levels of β-catenin and GAPDH (as a loading control) were evaluated by western blot analysis. We also examined total and active β-catenin levels after IWR-1 treatment for 16 h. **(B)** One-hundred twenty hours after siRNA introduction, cell numbers were evaluated as in Materials and Methods. Error bars represent standard deviation (SD) of three independent experiments. Statistical evaluation was done by Student *t* test (**: $P < 0.01$).

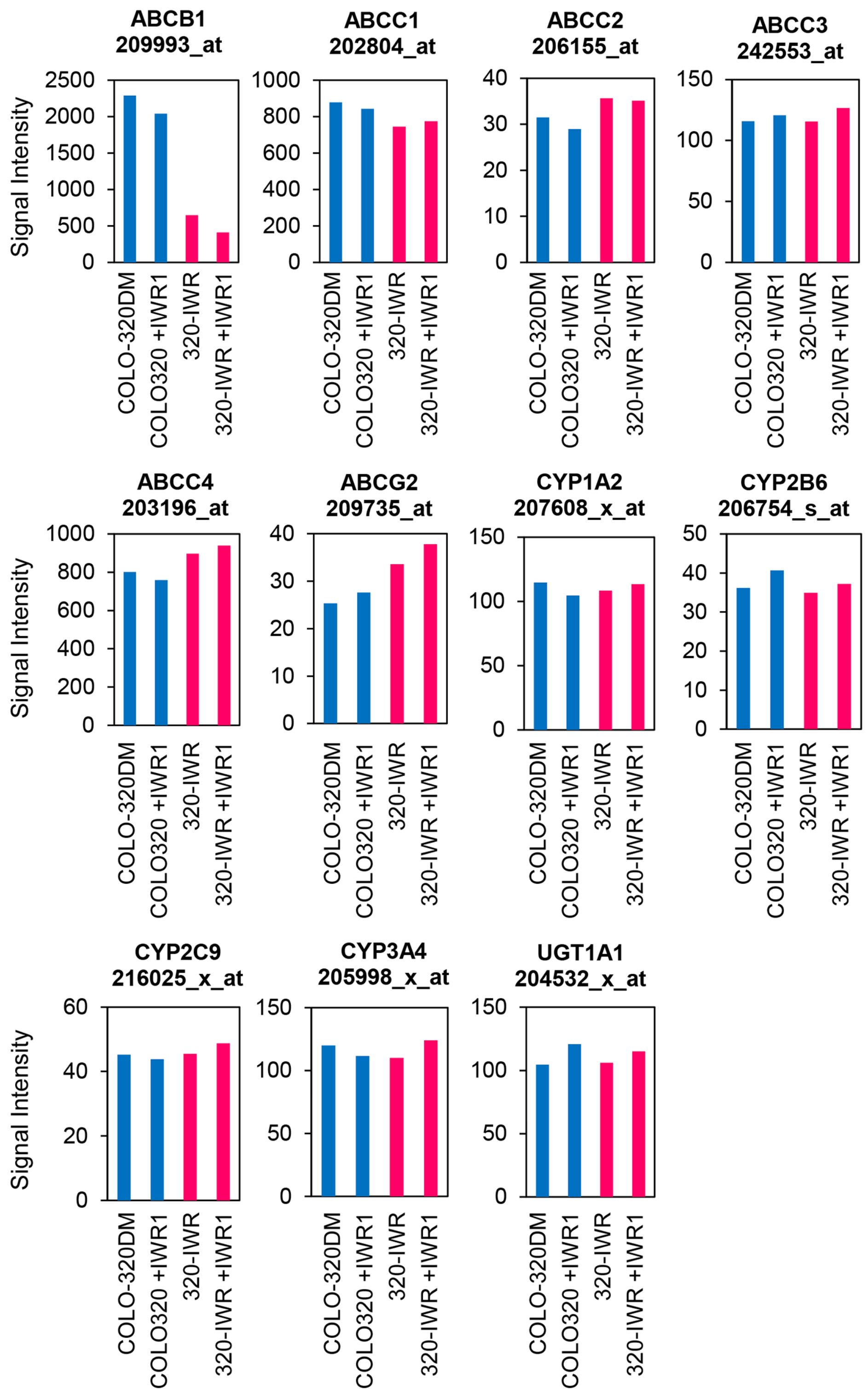

Supplementary Figure 6: Expression of genes involved in drug transport and drug metabolism in 320-IWR cells. From the cDNA microarray data of COLO-320DM and 320-IWR cells, we summarized the expression of genes related to drug transport and drug metabolism. Raw signal intensity values are shown.

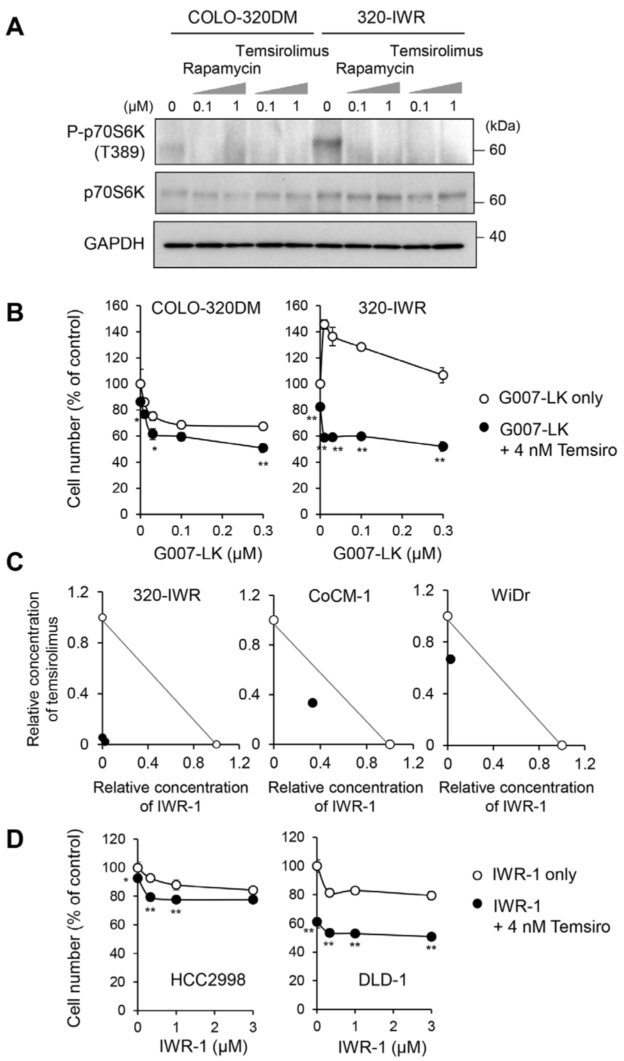

Supplementary Figure 7: Synergistic anti-proliferative effect of tankyrase inhibitor and mTOR inhibitor in colorectal cancer cells. **(A)** Effect of mTOR inhibitors temsirolimus and rapamycin on p70S6 kinase (p70S6K) in COLO-320DM and 320-IWR cells. Cells were treated with temsirolimus or rapamycin at the indicated concentrations for 2 h. Protein levels and phosphorylation status of p70S6K were evaluated by western blot analysis. **(B)** COLO-320DM and 320-IWR cells were treated with G007-LK and temsirolimus together at the indicated concentrations for 120 h. Cell numbers were evaluated as described in Materials and Methods. Error bars represent standard deviation (SD) of three independent experiments. Statistical significance was evaluated by Tukey-Kramer test (*: $P < 0.05$; **: $P < 0.01$). **(C)** Isobologram analysis was performed according to the calculation method as described [21]. In the graph, the combinational effect of two drugs is synergistic if the closed circle dot is left under the line. **(D)** HCC2998 and DLD-1 cells were treated with IWR-1 and temsirolimus at the indicated concentrations for 120 h. Cell numbers were determined as described in Materials and Methods. Error bars represent standard deviation (SD) of three independent experiments. Statistical significance was evaluated by Tukey-Kramer test (*: $P < 0.05$; **: $P < 0.01$).

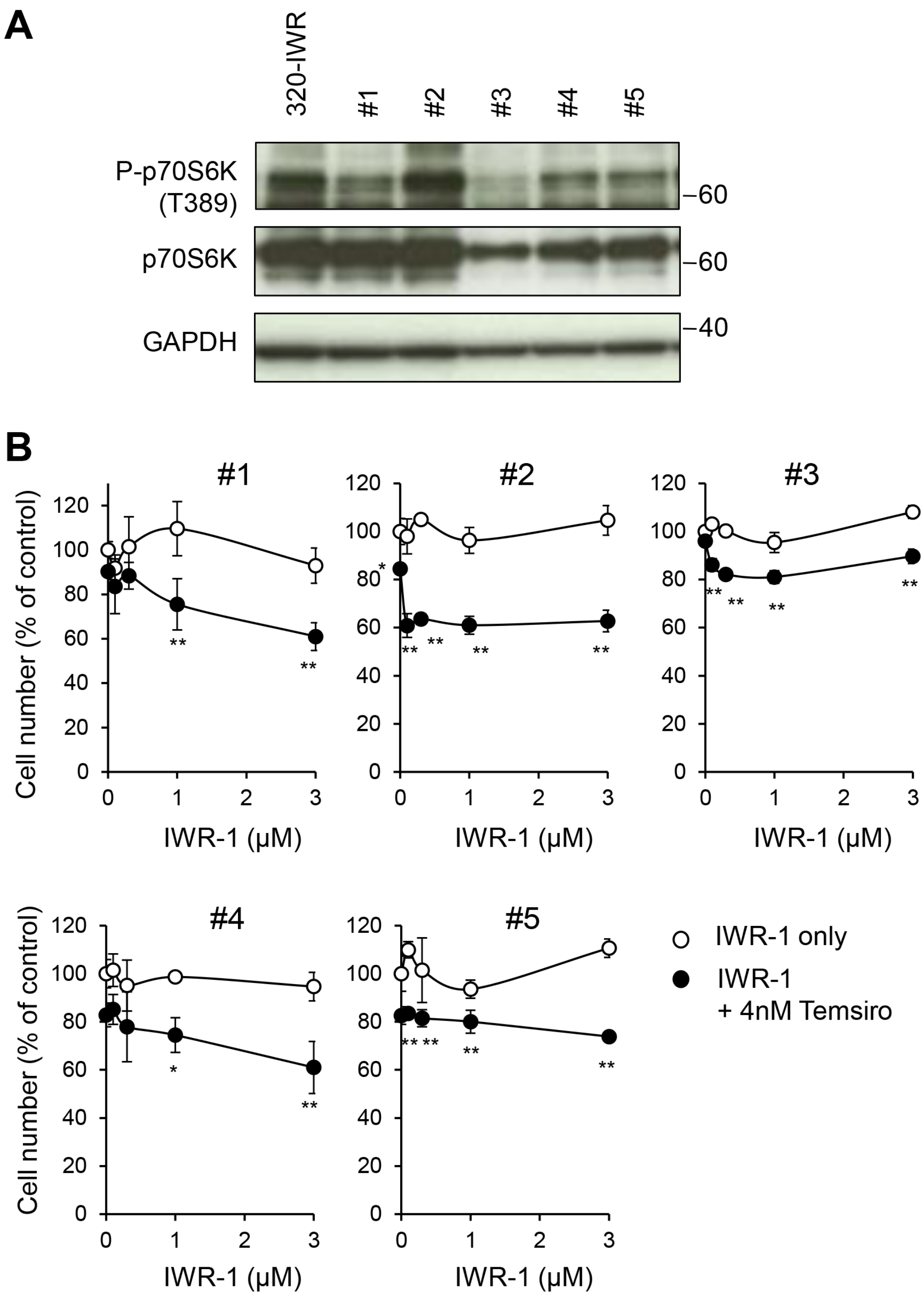

Supplementary Figure 8: Involvement of mTOR activation in tankyrase inhibitor-resistance of 320-IWR clones. (A) Phosphorylation level of mTOR pathway regulator, p70S6K in 320-IWR clones. Protein levels and phosphorylation status of p70S6K were evaluated by western blot analysis. **(B)** Reversal effect of temsirolimus on tankyrase inhibitor resistance of 320-IWR clones. Cells were treated with IWR-1 and temsirolimus (Temsiro) together at the indicated concentrations for 120 h. Cell numbers were calculated as described in Materials and Methods. Error bars represent standard deviation (SD) of three independent experiments. Statistical significance was evaluated by Tukey-Kramer test (*: $P < 0.05$; **: $P < 0.01$).

Supplementary Table 1: Cell cycle and apoptosis analysis in COLO-320DM and 320-IWR cells

Cells	Treatment	Sub-G1 (%)	G1 (%)	S (%)	G2/M (%)
COLO-320DM	No treatment	3.5	67.9	9.1	15.3
	IWR-1	2.5	65.5	8.7	16.6
	G007-LK	3.4	62.7	8.2	18.7
320-IWR	No treatment	5.5	56.9	4.5	21.2
	IWR-1	4.7	53.9	4	22.3
	G007-LK	6.2	52.3	5.3	21.4

NOTE: COLO-320DM and 320-IWR cells were treated with tankyrase inhibitors, IWR-1 (9 µM) or G007-LK (1 µM) for 120 h. Percentage of cells in each phase of the cell cycle and apoptotic cells (sub-G1 cells) were quantified by FACSCalibur flow cytometer.

Supplementary Table 2: Genes differentially expressed in 320-IWR cells.

See Supplementary File 2

Supplementary Table 3: Primers and probes for RT-qPCR

	Forward	Reverse	Probe No.
APCDD1	cgcctcctgctcagatacc	ggatgaaggagggcagaac	14
AXIN2	cacacccttctccaatccaa	tgccagtttctttggctctt	36
NKD1	tctcgccgggatagaaaac	gggggcagttctgacttct	36
MYC	gctgcttagacgctggattt	taacgttgaggggcatcg	66
LEF1	cagtcgacacttccatgtcc	atgagggatgccagttgtgt	79
JAG1	tgaccagaatggcaacaaaa	cagccttgtcggcaaatag	42
LGR5	accagactatgcctttggaaac	ttcccagggagtggattctat	78
BIRC5	gcccagtgtttcttctgctt	ccggacgaatgctttttatg	11
tankyrase-2	tgggtgtccagttcacaaag	aaggttacccggcaaaaga	27

NOTE: mRNA expression was evaluated by RT-qPCR with the indicated primers and probes using LightCycler 96 (Roche).
